# Changes in Chemical Composition of Flaxseed Oil during Thermal-Induced Oxidation and Resultant Effect on DSC Thermal Properties

**DOI:** 10.3390/molecules27207135

**Published:** 2022-10-21

**Authors:** Zhenshan Zhang, Yunyi Wei, Ni Guan, Bingzheng Li, Yong Wang

**Affiliations:** 1 College of Food Science and Engineering, Henan University of Technology, Zhengzhou 450001, China; 2 College of Food Science and Quality Engineering, Nanning University, Nanning 530200, China; 3 Guangxi Bioscience and Technology Research Center, Guangxi Academy of Sciences, Nanning 530007, China; 4 School of Chemical Engineering, UNSW, Sydney, NSW 2052, Australia

**Keywords:** flaxseed oil, thermal-induced oxidation, fatty acid composition, triacylglycerol profile, differential scanning calorimetry

## Abstract

To investigate the changes in chemical composition of flaxseed oil during thermal-induced oxidation and the resultant effect on thermal properties, samples with different oxidation levels were obtained by being heated at 180 °C for two hours and four hours. The oxidation degree was evaluated using peroxide value (PV), extinction coefficient at 232 nm and 268 nm (K_232_ and K_268_), and total polar compounds (TPC). Using chromatography, the fatty acid profile and triacylglycerol (TAG) profile were examined. Differential scanning calorimetry (DSC) was used to determine the crystallization and melting profiles. Thermal-induced oxidation of flaxseed oil led to a significant increase (*p* < 0.05) in PV, K_232_, K_268_, and TPC, but the relative content of linolenic acid (Ln) and LnLnLn reduced dramatically (*p* < 0.05). TPC derived from lipid degradation affected both crystallization and melting profiles. Statistical correlations showed that the onset temperature (*T_on_*) of the crystallization curve was highly correlated with K_232_, TPC, and the relative content of LnLnLn (*p* < 0.05), whereas the offset temperature (*T_off_*) of the melting curve was highly correlated with the relative content of most fatty acids (*p* < 0.05). This finding provides a new way of rapid evaluation of oxidation level and changes of chemical composition for flaxseed oils using DSC.

## 1. Introduction

The α-linolenic acid (ALA) is an essential fatty acid that cannot be synthesized by the human body and needs to be obtained from food. It can be converted into eicosapentaenoic acid (EPA) and docosahexaenoic acid (DHA), which are responsible for several important biological processes [1]. As a good source of polyunsaturated fatty acid, flaxseed oil contains approximately 55% ALA [2]. Hence, flaxseed oil is considered nutritionally healthy for human and is popular in many countries [3]. However, it is well known that the oxidation stability of vegetable oils is mainly associated with their unsaturated fatty acid composition. The high content in ALA makes flaxseed oil more susceptible to oxygen, light, and heat than other edible oils [4]. Besides, the oxidation process might also be affected by the structure and the physical state of the oils [5,6]. Lipid oxidation is usually responsible for the nutrition degradation and unpleasant flavor of edible oils. Moreover, many of the deterioration products generated during oxidation are harmful to human health [7]. Consequently, although flaxseed oil is recommended to be used at low temperatures, there are places where it is still used as a cooking and frying oil at up to 190 °C [8,9]. Therefore, it is crucial to precisely track the oxidative destruction of vegetable oils, particularly flaxseed oil, for the sake of human health.

There are numerous ways to evaluate the oxidative degradation of edible oils, including the peroxide value (PV), anisidine value (AnV), total polar compounds (TPC), and specific UV extinction coefficients (K_232_ and K_268_). In general, these approaches yield more precise answers, but they are time-consuming, labor-intensive, and necessitate the use of toxic chemicals that are hazardous to analysts and the environment [10]. Determining and monitoring the level of vegetable oil oxidation necessitates a quick, dependable, and instrumented technique.

Differential scanning calorimetry (DSC) is a physical analysis technique that does not require complex sample preparation and the use of any chemicals. It monitors the variation in heat flow resulting from phase transition and chemical reaction as a function of temperature or time [11,12]. DSC curves are characterized by endothermic and/or exothermic peaks whose area is proportional to the enthalpy gained or lost by the material undergoing phase transition [13]. On this basis, DSC thermograms of edible oils are derived from the thermal events of TAG molecules and minor ingredients present in the oils that overlap [14]. In the past decades, the DSC technique has been extensively used in the study of edible oils and is considered as a valuable tool to assess the deterioration of edible oils [15,16,17,18,19]. However, to the best of our knowledge, there are few reports analyzing the effect of changes in the chemical composition of vegetable oils during oxidation, such as fatty acids, triglycerides, and their oxidation products on the DSC properties of the oils, especially for flaxseed oil.

The objective of this study was to evaluate the effect of chemical composition of flaxseed oil during thermal-induced oxidation on its DSC properties. In this study, flaxseed oil samples with different oxidation levels were obtained by being heated at 180 °C (to simulate deep-frying temperature) for two hours and four hours. The changes in peroxide value (PV), UV extinction coefficients at 232 nm (K_232_) and 268 nm (K_268_), total polar compounds (TPC), fatty acid composition, TAG profile, and DSC thermograms (cooling and melting profiles) were determined. In addition, the correlations between thermal parameters and chemical compositions of flaxseed oil were also investigated.

## 2. Results and Discussion

### 2.1. Oxidation of Flaxseed Oil

In this work, the oxidation status of flaxseed oil was evaluated using PV, K_232_, K_268_, and TPC. The oxidation of vegetable oil is a complex process resulting in various decomposition products. In general, unsaturated fatty acids are oxidized into hydroperoxides, which are unstable under heating conditions and could readily decompose to secondary oxidation products, such as ketones, aldehydes, acids, alcohols, esters, and polymers [20]. The primary oxidation products, including hydroperoxides and conjugated dienes, are usually determined by PV and K_232_, whereas the secondary oxidation products could be accurately evaluated by K_268_, which is related to conjugated triene. TPC is an additional significant indicator for heated oils, representing all oxidation and breakdown products created during heating. As shown in Table 1, all oxidation parameters increased significantly (*p* < 0.05) with heating time increase. The PV, K_232_, K_268_, and TPC values of raw flaxseed oil were 6.64 meq O_2_/kg, 2.43, 0.45, and 5.04%, respectively, indicating the oil had an accepted quality [21]. After heating for 2 h, the PV and TPC increased sharply to 67.24 meq O_2_/kg and 30.78%, which exceeded the maximum permitted limits of PV (15 meq O_2_/kg) and TPC (25–27%) for edible oils [22]. The increment of PV and K_232_ during the second two hour heating was smaller than that which occurred in the first two hour heating, but an opposite tendency was found for K_268_. This phenomenon could be attributed to the decomposition of primary oxidation products as explained in previous studies [23]. In our investigation, the oxidation degree of flaxseed oil heated for four hours was equivalent to that of oil heated in a pan for three minutes at 150 °C [6], as well as those with oven-heating for 32 h at 135 °C [24]. This indicates that the oxidation rate of flaxseed oil was closely related to temperature and oil-oxygen contact area during thermal processing. Moreover, it has also been reported that oxidation can be retarded and restricted to a limited level when the oils are encapsulated in Pickering emulsions [6].

### 2.2. Fatty Acid Composition

As shown in Table 1, thermal-induced oxidation had a significant influence (*p* < 0.05) on the fatty acid composition of flaxseed oil. The fatty acids of raw flaxseed oil were mainly composed of linolenic acid (57.96%), followed by linoleic acid (17.48%), oleic acid (15.56%), palmitic acid (5.21%), and stearic acid (3.80%). The values are on a comparable level with the results reported in previous studies [25,26]. It can be found that there was a linear negative relation between linolenic acid content and heating time during accelerated oxidation. After heating for four hours, the relative content of linolenic acid decreased by 5.59%, whereas the relative content of palmitic, stearic, oleic, and linoleic acid accordingly increased by 1.14%, 1.02%, 2.86%, and 0.57%, respectively. The results are in agreement with the observation in previous studies, in which flaxseed oil was oxidized through pan-heating and oven-heating [8,24].

Fatty acids were grouped into saturated fatty acids (SFA), monounsaturated fatty acids (MUFA), and polyunsaturated fatty acids (PUFA) in terms of their unsaturation degree. During the oxidation process, there was a significant decrease in the relative content of PUFA (~5%), resulting in a concomitant increase in MUFA (~3%) and SFA (~2%). The majority of the decrease in PUFA can be attributed to their elimination by oxidation, which is in agreement with finding reported in previous study [27]. The oxidation of lipids may break the double bonds in PUFA into a single double bond (MUFA) and then into a single bond (SFA) [28]. The ratio of polyunsaturated to saturated fatty acids (P/S) is also a valid indication for assessing oil oxidation. In our investigation, the P/S ratio decreased as oxidation time increased. The results are consistent with previous report in groundnut oil by Ali, et al. [29].

### 2.3. Triacylglycerol Composition

**Figure 1:**
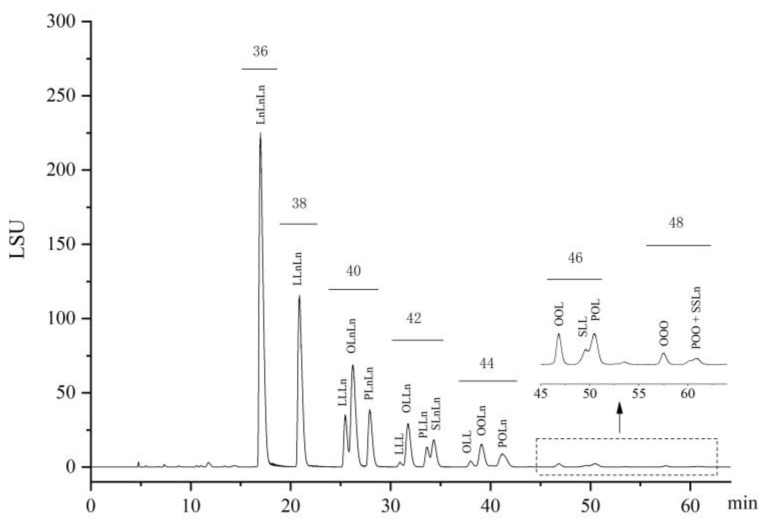
HPLC chromatogram of the triacylglycerol composition of raw flaxseed oil. Notes: LSU is the unit of signal response of the device when using an evaporative light scattering detector; the numbers above peaks represent the equivalent carbon number (ECN).

**Figure 2:**
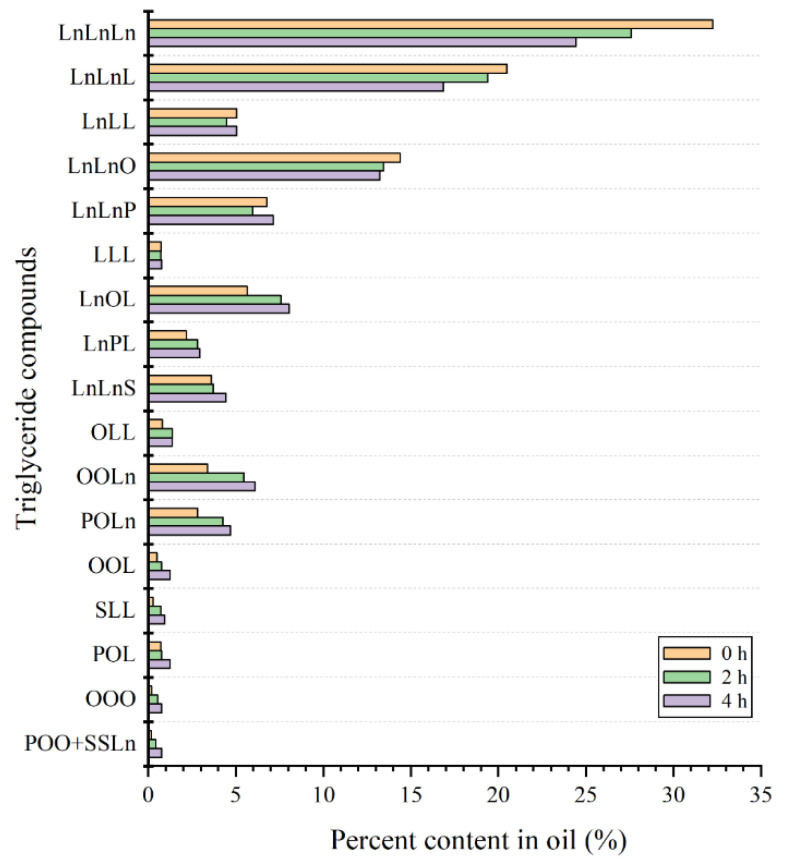
Changes of TAG composition of flaxseed oil during thermal-induced oxidation: P, Palmitic (C16:0); S, Stearic (C18:0); O, Oleic (C18:1); L, linoleic (C18:2); Ln, linolenic (C18:3).

The TAG composition of flaxseed oil was identified using the HPLC-ELSD method. A typical HPLC chromatogram of TAG composition of flaxseed oil is given in Figure 1. The value and change of TAG composition during thermal-induced oxidation are shown in Table 2 and Figure 2, respectively. In this study, eighteen TAG species were identified in all flaxseed oil samples. The dominant TAG in the raw flaxseed oil were LnLnLn (32.24%), followed by LnLnL (20.49%), LnLnO (14.38%), LnLnP (6.76%), LnOL (5.65%) and LnLL (5.06%). The results are in good agreement with previous report by Affes, et al. [30]. As anticipated, heating time had a significant impact on flaxseed oil’s TAG composition. With increasing heating time, the relative content of LnLnLn, LnLnL, and LnLnO declined significantly, whereas the percentage of all other TAG except LnLL, LnLnP, and LLL grew significantly. The percentages of LnLnLn, LnLnL, and LnLnO fell by 7.81%, 3.64%, and 1.16%, respectively, after four hours of heating. This may be because these TAG are predominantly made of polyunsaturated fatty acids, making them more unstable. In general, the rate of fatty acid breakdown is related to the number of double bonds in the carbon chain of the molecule. It was reported that the oxidation rate of oleate: linoleate: linolenate was 1:10:20 [31]. The oxidative stability of TAG could be indicated by equivalent carbon number (ECN). All TAG in flaxseed oil can be classified into seven types of ECN with a value from 36 to 48 (Figure 1). TAG below 40 ECN including LnLnLn (36 ECN), LnLnL (38 ECN) and LnLnO (40 ECN) were more susceptible to oxidation than those above 42 ECN in this study. Additionally, TAG existed in flaxseed oil can also be grouped into diunsaturated triacylglycerol (DUTAG) and triunsaturated triacylglycerols (TUTAG) according to the type of fatty acid bonded to the glycerol structure. After four hours heating, the content of TUTAG decreased by 5.59% (from 83.43% to 77.84%) with a corresponding increase in DUTAG.

### 2.4. Thermal Properties

#### 2.4.1. Cooling Profiles

**Figure 3:**
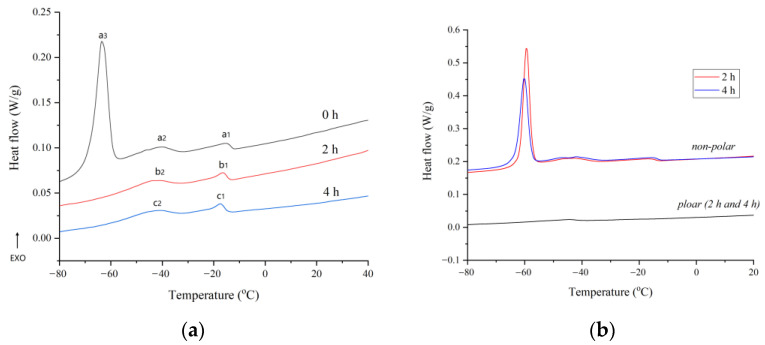
Cooling thermograms of flaxseed oil and its fraction at different heating times: (**a**) flaxseed oil; (**b**) polar and nonpolar fraction of flaxseed oil.

The DSC cooling profiles of unheated and heated flaxseed oil are reported in Figure 3a. The cooling curve parameters of flaxseed oil with different heating times are presented in Table 3. The crystallization profile of raw flaxseed oil (0 h) showed three well-distinguishable exothermic peaks, one major peak (peak a_3_) at lower temperature (*T_peak_* = −63.54 °C), and two minor peaks (peak a_1_ and peak a_2_) at higher temperature (*T_peak_* = −40.11 °C and −15.37 °C, respectively). The results are slightly different to the report given by Teh and Birch [32], in which only a distinct crystallization peak at −53.79 °C was found. This is possibly due to the larger heating rate used in this study. In general, faster heating rates result in larger peak areas and sharper peaks [33]. Thermal-induced oxidation made a great change in the cooling profile of flaxseed oil (Table 3). After two hours of heating, the prominent peak, which was mostly linked with the crystallization of highly unsaturated TAG, disappeared. The minor peaks, which were associated with more TAG saturation, migrated towards lower temperatures as heating time increased. Changes could be attributed to an increase in polar molecules resulting from the oxidative breakdown of lipids, which were supposed to interfere with TAG crystallization [34]. To validate this hypothesis, heated flaxseed oils were separated into polar fraction and nonpolar fraction using a silica column. Cooling profiles of the two fractions are shown in Figure 3b. The nonpolar fraction exhibited similar transition events with raw flaxseed oil, having a major peak and two minor peaks, but the major peak appeared at a higher temperature (*T_peak_* = −59.28 °C). This can be explained by the fact that lipid oxidation reduced the content of unsaturated fatty acid and TUTAG in the oil sample, and consequently reduced their percentage in the non-polar fraction [35]. In general, oil containing more unsaturated fatty acids crystallizes at a lower temperature than oil carrying more saturated fatty acids. On the cooling curve of the polar fraction there was essentially no thermal transition, with the exception of a faint peak about −45 °C. This behavior was also noticed in heated sunflower oil by Gloria and Aguilera [22]. The thermal parameters obtained from the cooling curves of all oil samples are reported in Table 3. The *T_on_* and exothermic enthalpy (*ΔH*) of cooling events decreased significantly with the increase of heating time (*p* < 0.05). This may be related to the partial cleavage of TAG and the formation of polar compounds that were absorbed into crystal lattices of TAG, forming irregular and weak crystals [36].

#### 2.4.2. Melting Profiles

**Figure 4:**
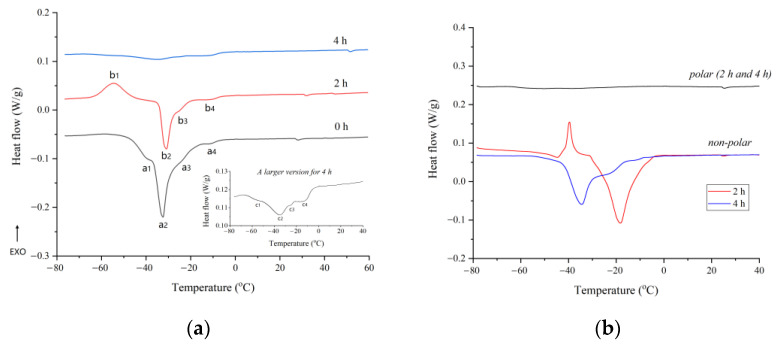
Heating thermograms of flaxseed oil and its fraction at different heating times: (**a**) flaxseed oil; (**b**) polar and nonpolar fraction of flaxseed oil. Note: the insert in Figure 4a is an enlarged plot of melting curves of flaxseed oil heated for four hours.

The DSC melting curves of unheated and heated flaxseed oil are reported in Figure 4. The melting curve parameters of flaxseed oil with different oxidation levels are presented in Table 3. Melting curves are generally more complex compared with cooling curves, because they originated from the overlapping of melting events of several crystal polymorphic forms [14]. As shown in Figure 4a, four endothermic events are observed on the melting curve of raw flaxseed oil (0 h), including a major peak (peak a_2_) and three shoulder peaks (peak a_1_, peak a_3_, and peak a_4_). The major endothermic event peaked at −32.43 °C, it was mainly attributed to the melting of TUTAG. The endothermic shoulder event at lower temperature (−38.39 °C) may be related to highly unsaturated TAG, whereas those at higher temperatures (−24.81 °C and −12.67 °C) may be associated with low unsaturated TAG [37]. On the melting profile of flaxseed oils, the duration of thermal-induced oxidation had a discernible effect. With increased heating time, the endothermic peaks became less distinct and broader until they eventually disappeared, corresponding to a decrease in *ΔH* and an increase in *ΔT* (Table 3). This is due to the decrease of unsaturated TAG and the creation of mixed crystals caused by the adsorption of lipid oxidation products into the crystal lattices of TAG [36]. It is worth noting that an exothermic event (peak b_1_) occurred on the melting curve of flaxseed oil heated for two hours. It is possibly due to the rearrangement of polymorphic crystals of oxidized TAG into more stable forms [36].

Figure 4b illustrates the heating thermograms of polar and nonpolar fractions isolated from heated flaxseed oils in order to verify the influence of polar compounds generated by lipid oxidation on the melting profile of flaxseed oil. On the melting curves of polar fraction, it was discovered that no observable thermal event appeared. This further demonstrated that these polar compounds were not susceptible to freezing within the temperature range examined [38]. The melting curve of nonpolar fraction derived from oil heated for four hours was comparable to that of oil samples, with the exception of a more pronounced shoulder peak at higher temperatures due to a greater concentration of saturated fatty acids in the oils. However, it is surprising that the melting curve of nonpolar fraction of oil heated for two hours exhibited two endothermic events peaking at −45.70 °C and −18.23 °C, respectively, and an exothermic event peaking at −39.53 °C, which was completely different with those obtained from raw flaxseed oil (0 h) and nonporlar fraction of oil heated for four hours. To the best of our knowledge, this may be the first confirmation that thermal-induced oxidation can fundamentally alter the thermal properties of the nonpolar fraction of oxidized oils, although previous studies have reported that deep-frying led to appreciable change in thermogram of vegetable oils [39].

### 2.5. Statistical Correlation between Chemical Composition and Thermal Properties

Table 4 summarizes the Pearson correlations between the oxidation indices, fatty acid composition, TAG profile, and thermal properties. Statistical analysis indicated that PV was significantly correlated (*p* < 0.05) with most of the thermal parameters in both cooling and heating thermograms. PV was positively correlated with *T_off_* and negatively correlated with *T_on_* and enthalpy value (*ΔH*). In addition, TPC and K_232_ exhibited a significantly (*p* < 0.05) negative correlation with *T_on_* upon cooling thermogram.

Statistical correlations between fatty acid composition and thermal parameters in cooling thermogram were not observed in this study. On the contrary, significant correlations were obtained between fatty acid composition and *T_off_* upon heating thermogram. In terms of TAG composition, the thermal parameters were mainly related to LnLnLn, LnLnO and LnOL. Table 4 presents that *T_on_* in cooling thermogram was strongly correlated with LnLnLn, whereas *ΔT* and the enthalpy of thermal events were strongly correlated with LnLnO and LnOL. Moreover, *T_off_* in the heating thermogram was significantly (*p* < 0.05) correlated with TUTAG and DUTAG. It is worth pointing out that not all TAGs affected the parameters on the DSC curves, as several pure TAGs did not show crystallization [40].

## 3. Materials and Methods

### 3.1. Materials

Flaxseed was purchased from a local market in Henan province, China. The seed was cleaned carefully to remove all foreign materials such as other seeds and stones. The cleaned seed was crushed into powder and stored in sealed plastic bags at −20 °C until use. Standard of fatty acid methyl esters (FAMEs) was purchased from Sigma-Aldrich Co. (Shanghai, China). All other chemicals and reagents were of analytical or chromatographic grade.

### 3.2. Oil Extraction by Cold Pressing

The flaxseed powder was pressed at ambient temperature using a hydraulic press (Zhengzhou Bafang Machinery Equipment Co., Ltd., Zhengzhou, China) under a constant pressure of 40 MPa for 30 min and repeated for 4 times. The collected oils were centrifuged at 5180× *g* for 10 min to remove suspended impurity and then stored at −10 °C until further analysis.

### 3.3. Oxidation of Flaxseed Oil

Fifty grams of flaxseed oil were poured into a 250 mL three-neck flask. The necks were connected with air, a mechanical stirrer, and a temperature sensor, respectively. The accelerated oxidation was performed on an oil bath at 180 °C (to simulate deep-frying temperature) for 2 h and 4 h, respectively, with stirring of 200 rpm. After heating, the oils were cooled down to room temperature, then kept in dark glass bottles at −20 °C until further analysis.

### 3.4. Peroxide Value

Peroxide value of oil sample was measured using AOCS Office method Cd 8-53 [41]. Briefly, the oil sample (2 g) was diluted with chloroform–acetic acid (2:3) mixture, then 1.0 mL of saturated potassium iodide was added, stirred for 30 s and kept in the dark for 3 min. Finally, 100 mL of distilled water and 1.0 mL of 1% (*w*/*v*) starch indicator were added, and the solution was titrated with 0.01 mol/L sodium thiosulfate. The result was expressed as milliequivalent of peroxide per kilogram oils (meq O_2_/kg).

### 3.5. Specific Extinction

K_232_ and K_268_ of flaxseed oil were determined using the method recommended by International Organization for Standardization [42] and carried on an ultraviolet spectrophotometer (Model T6, Beijing Purkinje General Instrument Co., Ltd., Beijing, China).

### 3.6. Total Polar Compounds (TPC)

The determination of total polar compounds was performed using preparative fast column chromatography, as previously described by An, et al. [43]. Briefly, one gram of oil was diluted with 8 mL petroleum ether and then injected into a flash silica column (particle size 40–60 μm). The nonpolar and polar compounds were eluted on a full-automatic edible oil polar compounds separation system (Tianjin Bonna-Agela Technologies Co., Ltd., Tianjin, China). The elution solvent consisted of petroleum ether and diethyl ether (87:13, *v*/*v*) for nonpolar components, whereas acetone and diethyl ether (40:60, *v*/*v*) were used for the polar component. The elution process lasted 11 min and 19 min for nonpolar and polar compounds, respectively, at a flow rate of 25 mL/min. The eluents were concentrated in a rotary evaporator at 60 °C. The residual solvent was furtherly removed using a vacuum drying oven at 40 °C. The polar and nonpolar fractions were collected and weighted, respectively.

### 3.7. Fatty Acid Composition

Fatty acid composition was determined according to the method as published recently [25]. Briefly, oil sample was saponified with methanolic NaOH, then methylated using BF_3_-methanol to produce FAMEs. The analysis of FAMEs was carried out on an Agilent 7890B gas chromatography (Agilent Technologies, Wilmingston, DE, USA), coupled to a HP-88 chromatographic column (100 m × 0.25 mm i.d., film thickness 0.2 μm), equipped with a flame ionization detector. The initial column oven temperature was 140 °C for 5 min, then raised to 240 °C at a rate of 4 °C /min and held for 10 min. The detector and injector temperatures were maintained at 280 °C and 240 °C, respectively. The sample (1.0 μL) was injected with a split ratio of 1:50. Nitrogen was used as carrier gas at a flow rate of 22 mL/min. FAMEs were identified by comparing their retention time to authentic standards and quantified as the area percentage of each peak.

### 3.8. Triacylglycerol Composition

The triacylglycerol composition of flaxseed oil was analyzed according to our previous reported method [44]. The analysis was performed on a Waters e2695 HPLC system (Waters Corporation, Milford, MA, USA) equipped with a Waters 2424 ELSD detector and a Lichrospher C18 column (250 mm × 4.6 mm, 5 μm; Merk Co. Ltd., Darmstadt, Germany). The oil sample for analysis was diluted to 1% (*w*/*v*) in acetone and filtered through a 0.45 μm membrane filter. Then, diluent of 10 μL was injected into HPLC system. The temperature of separation column was set at 30 °C. The mobile phase consisted of acetonitrile (A) and isopropanol (B), with a flow rate of 0.8 mL/min. The elution program used was as follow: from 0 to 30 min, 70% A/30% B (*v*/*v*); from 30 to 70 min, 60% A/40 % B (*v*/*v*); from 70 to 90 min, 55% A/45% B (*v*/*v*); from 90 to 95 min, 70% A/30% B (*v*/*v*). Both detector and nebulizer temperatures were maintained at 55 °C. The content of TAGs was calculated by using area normalization approach.

### 3.9. Thermal Analysis

The melting and cooling characteristics of flaxseed oil were determined using a Q20 differential scanning calorimeter (TA Instruments Ltd., New Castle, DE, USA). The oil sample (8 ± 1 mg) was weighted into an aluminum pan and sealed with cover in place. An empty aluminum pan was used as reference. The sample was equilibrated at 30 °C, then heated to 60 °C at 10 °C /min and held for 5 min to eliminate thermal history. Subsequently, the sample was cooled to −80 °C at 2 °C /min and held for 2 min to define the cooling profile. After that, the sample was heated to 60 °C at 2 °C /min to define the melting profile. Nitrogen was the purge gas and flowed at 50 mL/min. The temperature and heat flow calibrations were executed using indium (melting point = 156.6 °C, enthalpy = 28.45 J/g) and *n*-dodecane (melting point = −9.65 °C, enthalpy = 216.73 J/g) prior to experiment. Thermograms were analyzed with Universal Analysis 2000 software (Version 4.3A, TA instruments Ltd., New Castle, DE, USA) to obtain enthalpy (*ΔH*, J/g), peak temperature (*T_peak_*, °C), onset temperature (*T_on_*, °C), and offset temperature (*T_off_*, °C). *T_on_* and *T_off_* are defined as the intersection of baseline and tangent at the transition. The temperature range of transitions *(ΔT*, °C) was calculated as the difference between *T_on_* and *T_off_*. The thermal characteristics of nonpolar and polar fractions separated from heated oils were also analyzed under the above conditions.

### 3.10. Statistical Analysis

All experiments were performed in triplicate and the results were expressed as means ± standard deviation. The one-way analysis of variance (ANOVA) and Duncan’s multiple comparison tests at a 95% confidence level (*p* < 0.05) were employed to determine the significant differences among mean values. Pearson correlation test was used to determine the correlations among data obtained. All statistical analyses were performed using SPSS 13.0 (SPSS inc., Chicago, IL, USA) statistical software.

## 4. Conclusions

This investigation confirmed that the fatty acid composition, TAG profile, and thermograms of flaxseed oil changed significantly as oxidation levels increased during thermal-induced oxidation. Linolenic acid is the predominant fatty acid in flaxseed oil, whereas LnLnLn, LnLnL, and LnLnO were the most abundant TAG molecules. All of their relative content decreased as the heating time increased. During thermal-induced oxidation, polar molecules interfered with the crystallization and melting of TAG groups, reducing the peaks and enthalpies of thermal transitions. In terms of TAG composition, statistical research revealed that the thermal parameters of flaxseed oil were primarily related to LnLnLn, LnLnO, and LnOL.

## Figures and Tables

**Table 1:** Changes in oxidation indices and fatty acid composition (%) of flaxseed oils during heating at 180 °C.

Indices	Heating Time (h)		
	0	2	4
Peroxide value (meq O_2_/kg)	6.64 ± 0.76 ^c^	67.24 ± 0.88 ^b^	76.80 ± 1.42 ^a^
K_232_	2.43 ± 0.15 ^c^	24.55 ± 0.08 ^b^	35.95 ± 0.11 ^a^
K_268_	0.45 ± 0.03 ^c^	4.90 ± 0.03 ^b^	14.68 ± 0.07 ^a^
TPC (%)	5.04 ± 1.15 ^c^	30.78 ± 2.30 ^b^	52.45 ± 1.78 ^a^
Fatty acids (relative content, %)			
C16:0 (palmitic acid)	5.21 ± 0.02 ^c^	5.65 ± 0.01 ^b^	6.35 ± 0.10 ^a^
C18:0 (stearic acid)	3.80 ± 0.13 ^c^	4.27 ± 0.05 ^b^	4.82 ± 0.04 ^a^
C18:1 (oleic acid)	15.56 ± 0.14 ^c^	16.73 ± 0.04 ^b^	18.42 ± 0.17 ^a^
C18:2 (linoleic acid)	17.48 ± 0.04 ^b^	17.78 ± 0.05 ^ab^	18.05 ± 0.15 ^a^
C18:3 (linolenic acid)	57.96 ± 0.34 ^a^	55.58 ± 0.04 ^b^	52.37 ± 0.45 ^c^
SFA	9.00 ± 0.16 ^c^	9.92 ± 0.04 ^b^	11.17 ± 0.13 ^a^
MUFA	15.56 ± 0.14 ^c^	16.73 ± 0.04 ^b^	18.42 ± 0.17 ^a^
PUFA	75.44 ± 0.30 ^a^	73.36 ± 0.01 ^b^	70.42 ± 0.30 ^c^
P/S	8.38 ± 0.18 ^a^	7.40 ± 0.03 ^b^	6.31 ± 0.10 ^c^

Notes: SFA, saturated fatty acid; MUFA, monounsaturated fatty acid; PUFA, polyunsaturated fatty acid; P/S, ratio of polyunsaturated to saturated fatty acid; values represent mean ± standard deviation of triplicates (*n* = 3); the one-way analysis of variance (ANOVA) and Duncan’s multiple comparison tests at a 95% confidence level were employed to determine the significant differences among mean values; different superscript letters in the same row indicate significant differences (*p* < 0.05) among different heating times.

**Table 2:** Changes in TAG profile of flaxseed oil during heating at 180 °C.

TAG Composition (Relative Content, %)	Heating Time (h)		
	0	2	4
LnLnLn	32.24 ± 0.35 ^a^	27.59 ± 0.70 ^b^	24.43 ± 0.91 ^c^
LnLnL	20.49 ± 0.13 ^a^	19.39 ± 0.21 ^b^	16.85 ± 0.43 ^c^
LnLL	5.06 ± 0.09 ^a^	4.48 ± 0.04 ^b^	5.05 ± 0.18 ^a^
LnLnO	14.38 ± 0.11 ^a^	13.44 ± 0.15 ^b^	13.22 ± 0.44 ^b^
LnLnP	6.76 ± 0.07 ^b^	5.96 ± 0.03 ^c^	7.14 ± 0.15 ^a^
LLL	0.73 ± 0.00 ^a^	0.71 ± 0.04 ^a^	0.76 ± 0.06 ^a^
LnOL	5.65 ± 0.01 ^c^	7.56 ± 0.08 ^b^	8.05 ± 0.18 ^a^
LnPL	2.19 ± 0.01 ^c^	2.80 ± 0.05 ^b^	2.94 ± 0.06 ^a^
LnLnS	3.60 ± 0.03 ^b^	3.72 ± 0.04 ^b^	4.44 ± 0.10 ^a^
OLL	0.80 ± 0.02 ^b^	1.38 ± 0.01 ^a^	1.36 ± 0.04 ^a^
OOLn	3.39 ± 0.05 ^c^	5.47 ± 0.10 ^b^	6.11 ± 0.13 ^a^
POLn	2.83 ± 0.08 ^c^	4.28 ± 0.01 ^b^	4.70 ± 0.10 ^a^
OOL	0.49 ± 0.01 ^c^	0.77 ± 0.03 ^b^	1.25 ± 0.03 ^a^
SLL	0.29 ± 0.04 ^c^	0.72 ± 0.03 ^b^	0.94 ± 0.02 ^a^
POL	0.71 ± 0.05 ^b^	0.76 ± 0.04 ^b^	1.25 ± 0.03 ^a^
OOO	0.20 ± 0.02 ^c^	0.55 ± 0.03 ^b^	0.76 ± 0.01 ^a^
POO + SSLn	0.18 ± 0.02 ^c^	0.43 ± 0.03 ^b^	0.75 ± 0.01 ^a^
TUTAG	83.43 ± 0.17 ^a^	81.34 ± 0.13 ^b^	77.84 ± 0.48 ^c^
DUTAG	16.56 ± 0.28 ^c^	18.67 ± 0.14 ^b^	22.16 ± 0.47 ^a^

Notes: values represent mean ± standard deviation of triplicates (*n* = 3). The one-way analysis of variance (ANOVA) and Duncan’s multiple comparison tests at a 95% confidence level were employed to determine the significant differences among mean values; different superscript letters in the same row indicate significant differences (*p* < 0.05) among different heating times.

**Table 3:** DSC data obtained from the thermograms of heated flaxseed oil.

Parameters	Heating Time (h)		
	0	2	4
*Cooling curves*			
*T_peak 1_* (°C)	−15.30 ± 0.11 ^a^	−16.50 ± 0.06 ^b^	−17.70 ± 0.35 ^c^
*T_peak 2_* (°C)	−40.45 ± 0.47 ^a^	−41.55 ± 0.68 ^a^	−40.90 ± 0.26 ^a^
*T_peak 3_* (°C)	−63.66 ± 0.17	-	-
*T_on_* (°C)	−12.39 ± 0.01 ^a^	−13.63 ± 0.30 ^b^	−14.46 ± 0.07 ^c^
*T_off_* (°C)	−69.15 ± 0.17 ^b^	−61.13 ± 2.21 ^a^	−60.40 ± 2.33 ^a^
*ΔT* (°C)	56.77 ± 0.18 ^a^	47.50 ± 2.52 ^b^	45.94 ± 2.40 ^b^
*ΔH* (J/g)	42.22 ± 0.38 ^a^	9.50 ± 0.02 ^b^	8.84 ± 1.03 ^b^
*Melting curves*			
*T_peak 1_* (°C)	−38.39 ± 0.04 ^a^	−54.47 ± 0.07 ^b^	−54.33 ± 0.11 ^b^
*T_peak 2_* (°C)	−32.43 ± 0.01 ^a^	−30.94 ± 0.05 ^a^	−34.77 ± 1.15 ^b^
*T_peak 3_* (°C)	−24.81 ± 0.30 ^a^	−25.38 ± 0.50 ^a^	−25.41 ± 0.01 ^a^
*T_peak 4_* (°C)	−12.67 ± 0.81 ^a^	−12.06 ± 0.24 ^a^	−12.94 ± 0.33 ^a^
*T_on_* (°C)	−48.02 ± 0.52 ^a^	−62.77 ± 0.01 ^b^	−64.30 ± 0.62 ^c^
*T_off_* (°C)	−6.93 ± 0.04 ^a^	−6.27 ± 0.13 ^a^	−5.35 ± 0.88 ^a^
*ΔT* (°C)	41.09 ± 0.48 ^b^	56.50 ± 0.14 ^a^	58.95 ± 1.50 ^a^
*ΔH* (J/g)	55.97 ± 0.45 ^a^	19.80 ± 0.57 ^b^	12.42 ± 0.13 ^c^

Notes: Values represent mean ± standard deviation of triplicates (*n* = 3); the one-way analysis of variance (ANOVA) and Duncan’s multiple comparison tests at a 95% confidence level were employed to determine the significant differences among mean values; different superscript letters in the same row indicate significant differences (*p* < 0.05) among different heating times.

**Table 4:** Pearson correlation coefficients between main chemical indices and thermal properties of flaxseed oil.

	Cooling Thermogram				Heating Thermogram			
	*T_on_* (°C)	*T_off_* (°C)	*ΔT* (°C)	*ΔH* (J/g)	*T_on_* (°C)	*T_off_* (°C)	*ΔT* (°C)	*ΔH* (J/g)
Oxidation indices								
PV (meq O_2_/kg)	−0.960	0.999 *	−1.000 **	−0.994	−0.999 *	0.881	1.000 **	−0.999 *
TPC (%)	−0.998 *	0.922	−0.943	−0.897	−0.925	0.990	0.940	−0.951
K_232_	−0.998 *	0.965	−0.979	−0.948	−0.967	0.962	0.977	−0.983
K_268_	−0.947	0.789	−0.824	−0.752	−0.795	0.993	0.820	−0.838
Fatty acid composition (relative content, %)								
C18:3	0.980	−0.861	0.889	0.830	0.866	−1.000 **	−0.886	0.900
C18:2	−0.997	0.914	−0.936	−0.889	−0.918	0.992	0.934	−0.945
C18:1	−0.976	0.851	−0.880	−0.819	−0.856	1.000 **	0.877	−0.892
C18:0	−0.987	0.881	−0.907	−0.852	−0.885	0.999 *	0.904	−0.917
C16:0	−0.970	0.837	−0.867	−0.804	−0.842	0.999 *	0.864	−0.880
SFA	−0.980	0.860	−0.888	−0.829	−0.865	1.000 **	0.885	−0.899
PUFA	0.978	−0.854	0.883	0.823	0.859	−1.000 **	−0.880	0.895
TAG composition (relative content, %)								
LnLnLn	1.000 **	−0.943	0.961	0.922	0.946	−0.979	−0.959	0.968
LnLnL	0.943	−0.782	0.817	0.745	0.788	−0.992	−0.813	0.831
LnLL	0.129	−0.447	0.394	0.498	0.438	0.080	−0.400	0.371
LnLnO	0.974	−0.995	0.999 *	0.987	0.996	−0.905	−0.999 *	1.000 *
LnLnP	−0.206	−0.127	0.069	0.184	0.117	0.404	−0.076	0.044
LnOL	−0.977	0.993	−0.998 *	−0.984	−0.994	0.911	0.998 *	−0.999 *
TUTAG	0.967	−0.829	0.860	0.796	0.835	−0.999 *	−0.857	0.873
DUTAG	−0.968	0.831	−0.862	−0.798	−0.837	0.999 *	0.859	−0.875

Note: Pearson correlation test was used to determine the correlations among data obtained. * Significance at the 0.05 level (*p* < 0.05); ** Significance at the 0.01 level (*p* < 0.01).

## Data Availability

The datasets generated and analyzed during the current study are available from the corresponding author on reasonable request.

## Sample Availability

Samples of the compounds are available from the authors.
